# Alternative Test Models: Ocular Safety Assays Accepted

**DOI:** 10.1289/ehp.116-a381

**Published:** 2008-09

**Authors:** Ernie Hood

In a significant step forward for alternative safety test methods designed to reduce, refine, or replace the use of live test animals, the Interagency Coordinating Committee on the Validation of Alternative Methods (ICCVAM) recently announced the regulatory acceptance of two new *in vitro* ocular safety assays by the U.S. Food and Drug Administration, Environmental Protection Agency, and Consumer Product Safety Commission. The acceptance was based on recommendations made by ICCVAM after an extensive evaluation of the methods.

The United States tallies an estimated 125,000 eye injuries in the home each year caused by accidental exposure to common household products such as bleach and oven cleaner, according to the American Academy of Ophthalmology. Proper identification and labeling of substances that can damage the eye is one way to combat such injuries. Several agencies require manufacturers to test new products for their potential to cause temporary or permanent blindness, irritation, or other eye injuries.

Working with the National Toxicology Program Interagency Center for the Evaluation of Alternative Toxicological Methods (NICEATM), ICCVAM evaluated and recommended the bovine corneal opacity and permeability (BCOP) and the isolated chicken eye (ICE) test methods—the first nonanimal ocular safety test methods to be accepted by the regulators. In both cases, the animal eyes used for the tests are slaughterhouse waste, so no animals are euthanized specifically to obtain these tissues. The assays have been in development since the early 1990s.

Now that the BCOP and ICE assays have earned regulatory acceptance, they must be considered as the first option for ocular safety testing under the Animal Welfare Act, which requires the consideration of alternative methods before animals are used for procedures that may cause more than slight or momentary pain or distress. “If you get a positive result in either of these assays, you can use that as a positive for the purposes of classifying and labeling [a material] as a severe irritant,” says Marilyn Wind, chair of ICCVAM and a deputy associate executive director with the Consumer Product Safety Commission. “If it’s negative, then [manufacturers] have to go to the next step and test in animals. This eliminates the most corrosive and severe chemicals from having to be tested in animals, so there is a reduction in potential pain and distress.”

Although precise numbers are not available for the use of live animals in ocular testing, William Stokes, director of NICEATM and executive director of ICCVAM, estimates that based on the relative distribution of adverse effects, use of the two assays could reduce the use of live animals for eye safety testing by 10% or more. “The overall goal is to come up with an integrated testing strategy using several nonanimal tests that will accurately predict whether chemical products have the potential to damage the eye or not,” he says. ICCVAM and NICEATM are in the process of evaluating other *in vitro* methods for ocular safety, hoping to eventually eliminate altogether the need for *in vivo* testing in this realm.

In the near term, ICCVAM is working with its counterparts in Europe and Japan to expedite approval of the BCOP and ICE assays at the international level by the 30-member Organisation for Economic Co-operation and Development. This group includes the United States, Canada, Japan, and most of the European Union, where a ban on live animal testing of cosmetic ingredients takes effect in March 2009 and the newly implemented REACH (Registration, Evaluation, Authorisation and Restriction of Chemical Substances) legislation will require testing of thousands of chemicals by 2018.

